# Natalizumab-associated progressive multifocal leukoencephalopathy (PML) in multiple sclerosis (MS): “a case report from Ireland with review of literature, clinical pitfalls and future direction”

**DOI:** 10.1186/s41983-020-00260-6

**Published:** 2021-01-07

**Authors:** Salman Mansoor, Gerard Mullane, Mohammad Hijaz Adenan, Siobhan Kelly, Aine Water, Grainne McPartland, Kevin Murphy

**Affiliations:** Department of Neurology, Sligo University Hospital, Sligo, Ireland

**Keywords:** Natalizumab, Progressive multifocal leukoencephalopathy, Cidofovir, Mirtazapine, Steroids, Multiple sclerosis

## Abstract

**Background:**

Progressive multifocal leukoencephalopathy (PML) is one of the most serious treatment-related complications that is encountered in patients with multiple sclerosis (MS). PML is a serious complication of MS treatment which is most commonly related to natalizumab.

**Case presentation:**

We report clinical course of progressive multifocal leukoencephalopathy (PML) in a 40-year-old man who was on treatment for highly active relapsing-remitting multiple sclerosis with natalizumab (Nz). He was treated with steroids, cidofovir, and mirtazapine and went on to develop long-term disability. The case describes the evolution of PML from diagnosis up till 5 months with changes on sequential brain scans and clinical symptoms in our patient.

**Conclusion:**

Patients who are on natalizumab should be aware and consented for the risk of PML. They should be periodically re-assessed for their relative PML risk. There is a growing body of evidence that suggests switching patients from natalizumab who have a higher risk of PML to other safer treatment options.

## Background

PML is a serious demyelinating disorder that is caused by John Cunningham virus (JCV) due to its reactivation in certain immune states in different disease conditions. It is exclusively a disease of immunosuppressed individuals, and various associations include malignancy, HIV infection, organ transplantation, and autoimmune disorders [1–3].

The antibodies to JCV are prevalent in almost 86% of adults after an initial asymptomatic infection with JCV in childhood [4]. The latent virus usually stays in the kidneys and lymphoid organs, but cellular immunosuppression can cause its reactivation. Viral replication under immunomodulation leads to production of neurotropic variants that can replicate in glial cells [1].

PML should be suspected in patients with new neurological symptoms or deterioration who are on immunomodulation. The diagnostic modalities that help establish the diagnosis include MRI-brain and cerebrospinal fluid analysis for the presence of JCV DNA by PCR.

Pembrolizumab and nivolumab are newer checkpoint inhibitors that have shown some good outcomes in small selected number of PML patients although larger studies are needed to establish their clinical efficacy [5–7].

PML is one of the most serious treatment-related complications that is encountered in patients with multiple sclerosis (MS) [8]. It poses a significant risk of long-term disability and mortality.

Natalizumab has been conventionally associated with PML in MS patients; however, other disease-modifying therapies which pose PML risk include rituximab, fingolimod, dimethyl-fumarate [9–15].

## Case presentation

We report a case of a 40-year-old man who presented with 7 days history of visual disturbance, leg weakness, pain, and unsteadiness. He reported seeing “spots” in his visual fields, double vision, and some retro-ocular pain. He described burning pain in his right thigh and proximal weakness of his right leg which restricted his daily activities. However, he was fully independent on presentation.

**Fig. 1:**
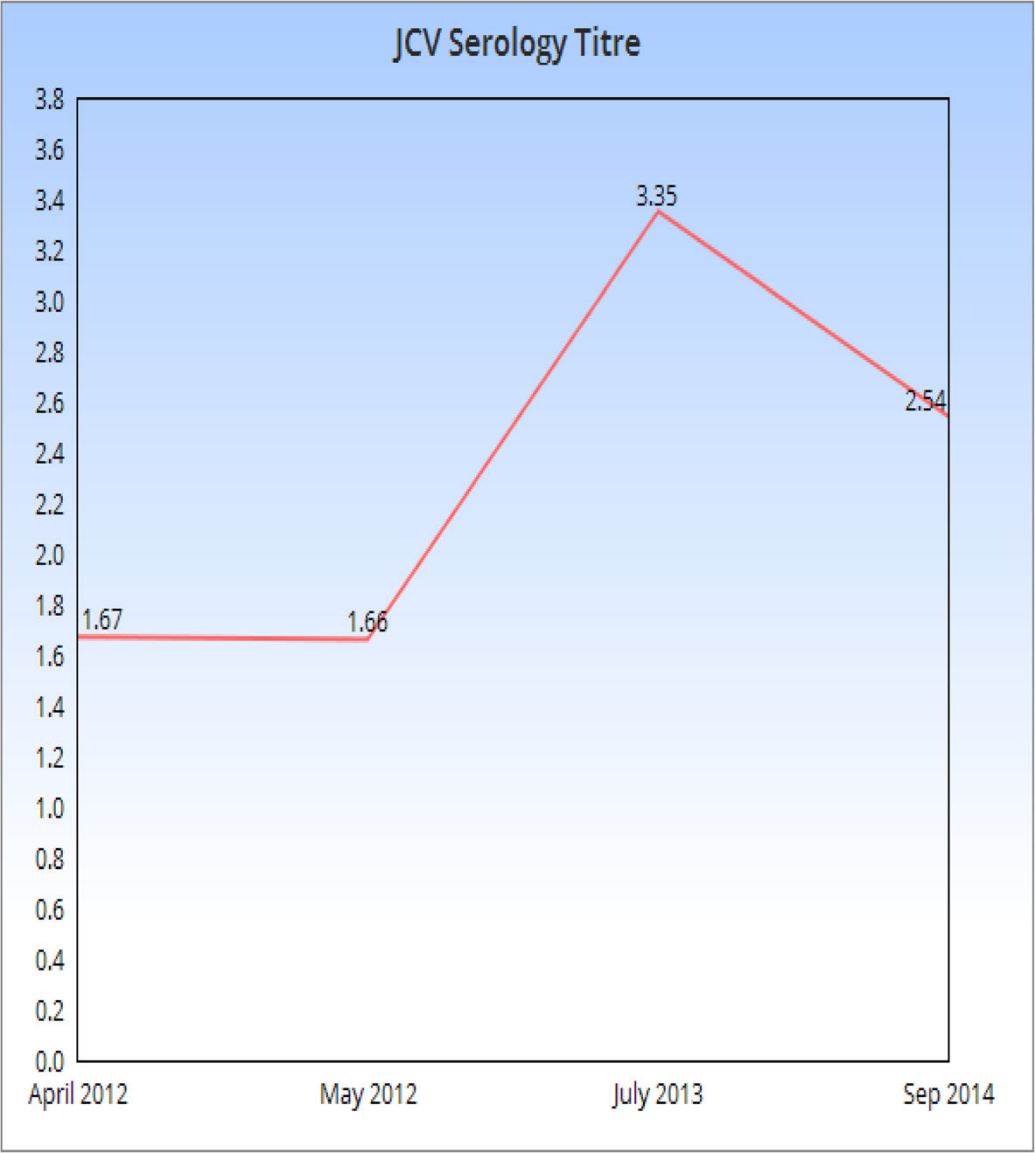
JCV serology index (2012–2014)

He had a medical history of relapsing-remitting multiple sclerosis for the last 15 years for which he was currently on monthly natalizumab for the last 9 years and received a total of 107 infusions over this period. Past disease-modifying therapies for multiple sclerosis (MS) included beta interferon and glatiramer acetate which were switched due to recurrent relapses. His serology for John Cunningham virus (JCV) was positive since 2012 as shown in Fig. 1.

He also had epilepsy for last 10 years, with a seizure semiology of partial with secondary generalized tonic-clonic seizures. He was on 4 anti-epileptics: levetiracetam 4gm/day, lamotrigine 600 mg/day, carbamazepine 700 mg/day, phenobarbitone 105 mg/day. The other regular medications included fesoterodine 8 mg, pyridoxine 100 mg, and vitamin D 2400 units daily.

His pertinent examination findings were horizontal nystagmus with diplopia in central and right gaze without any restriction in the eye movements. The right leg had reduced power grade in hip adduction, abduction, and flexion 4/5. His reflexes in bilateral lower limbs were 3+ with bilateral extensor plantar responses. There was past pointing on the right finger to nose test.

**Fig. 2:**
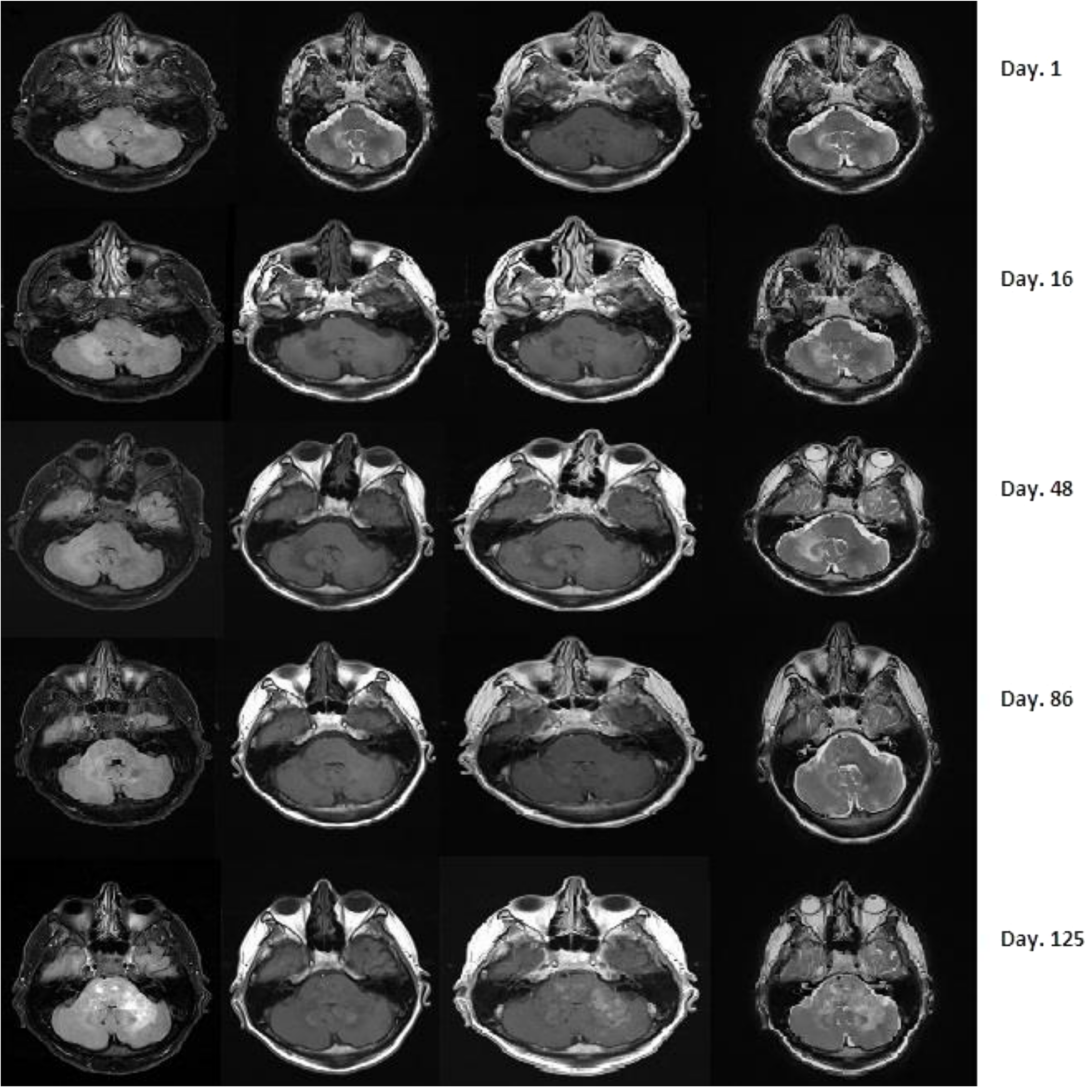
Showing MRI brain axial sequences FLAIR/T2/T1 and T1 with contrast at various stages. Day 1, new non-enhancing cerebellar hemisphere T2 hyperintensities (larger on the right). The new ill-defined extensive T2 hyperintensity in the right middle cerebellar peduncle extending into the right cerebellar white matter is felt concerning for PML. Day 16, no pathological enhancement identified. The previously noted new high signal in the right cerebellar peduncle extending into the right cerebellar hemisphere is slightly more extensive than on the prior scan but no enhancement. Day 48, progression of right cerebellar and brainstem high signal and enhancement. Progression of the presumed PML in the right middle cerebellar peduncle extending into the right cerebellar hemisphere and now extending to the right side of the pons. Day 86, area of abnormal T2 high signal within the right middle cerebellar peduncle extending into the pons and right cerebellum is again demonstrated. There is increased T2 signal at the periphery of the lesion within the right cerebellar hemisphere, and involvement of the pons is also significantly increased, now extending to involve the central pons. New area of T2 low signal within the medial left cerebellar hemisphere and in the left cerebellum adjacent to the inferior left cerebellar peduncle faint peripheral enhancement in the area of increased FLAIR signal in the right cerebellar hemisphere**.** Faint enhancement within the peripheral aspect of the right cerebellar lesion suggestive of PML-IRIS. MRI findings are consistent with progression of disease with progressive involvement of the pons and new involvement of the left cerebellar hemisphere. Day 125, significant progression of high signal abnormality in the posterior fossa as described, now extending to the left of the midline associated with extensive patchy enhancement. The appearances would now be more in keeping with PML-IRIS. (These MRI-images are acquired through 1.5 T Machine)

It was suspected that his current symptoms may be due to MS relapse. He was started on intravenous methyl prednisolone 1 g/day, and MRI brain was done as shown in Fig. 2 (day 1). The MRI brain prompted further investigation due to the atypical location and radiological features of the lesion including a lumbar puncture and CSF analysis for JCV-DNA PCR. Results received 3 days later showed a JCV viral load of 6620 copies/ml. Another lumbar puncture was done to further confirm the results 5 days that showed a viral load of 48,300 copies/ml.

He was started on mirtazapine at 60 mg daily doses. Cidofovir 5 mg/kg per dose, two doses 1 week apart followed by fortnightly doses a total of 6 doses over 12 weeks were administered. Mefloquine was considered but due to the potential of causing seizures in our patient was not commenced.

The clinical course is summarized in Table 1 over a 6 months period from PML onset to his transfer to a rehabilitation facility. Over this course, he deteriorated from being functionally independent to being wheel chair bound. There was a major deterioration in weeks 7–15 before his clinical condition stabilized and later improved with a major morbidity.

**Table 1:** Disease course from onset to the final outcome

Hospital course	Event	Clinical features	Disease evolution on MRI brain	Treatment	Outcomes
Day 1	Visual disturbance, leg weakness, pain, and unsteadiness	• Right-sided hip weakness • Ataxia • Diplopia and nystagmus	PML^x^ related changes in cerebellum	Methyl prednisolone 5 days, cidofovir, mirtazapine, probenecid	deterioration
Day 16	Headaches, worsening blurry vision, and diplopia	• Symptoms improved next 2 days • Mobility slightly improved	PML-related changes slightly more extensive	No changes made	Stable
Day 48	Prolonged generalized tonic-clonic seizure which lasted almost 50-min	• Decreased consciousness and right-sided Todd’s paresis. • Mechanical ventilation, ICU admission	Progression of PML	Loaded with phenobarbitone, methyl prednisolone 5 days, and oral taper over next 2 weeks	Deterioration
Day 86	Worsened swallow, weak cough reflex, and difficulty clearing secretions, requiring suctioning	• Significant dysarthria • Right hand weakness and impaired coordination • Gradually worsening swallow and gait, now using wheelchair • Bladder dysfunction	Progression of PML changes and features suggestive of worsening PML- and onset of IRIS^xx^	Started on 5/7 course of IV immunoglobulins, antibiotics for recurrent aspirations, PEG tube inserted for feeding	Deterioration
Day 125	Seizure, eye flickering, and unresponsiveness, self-aborted less than a minute	• Nausea and vomiting • Dysarthria started improving • Truncal ataxia, completely wheel chair bound	Significant progression of PML and IRIS	IV methyl prednisolone 1 g for 5 days given followed by oral prednisolone taper over 2 weeks	Improvement
Day 158	Continued to improve mobility, still limited to wheelchair but sitting balance significantly improved	• Speech became clearer • Truncal ataxia improved • Swallowing improved	MRI not done	Transferred for long-term rehabilitation, stable at this point	Improvement

^x^Progressive multifocal leukoencephalopathy

^xx^Immune reconstitution inflammatory syndrome

## Discussion

The risk of PML in patients who are on natalizumab have been linked to the number of infusions and the presence of anti-JCV in the serum [16]. The risk has been estimated to be less than 0.07 in 1000 patients who are anti-JCV negative, 1.7% in those who were anti-JCV positive, and 2.7% in those who were anti-JCV positive with previous immunosuppression use over a 6-year cumulative period (72 infusions of natalizumab) [16].

The current national guidelines in Ireland for checking JCV status in serum on patients who are to be started on natalizumab include baseline antibody test before commencing treatment and in those who are negative to be repeated 6-monthly [https://www.hse.ie/eng/about/who/acute-hospitals-division/drugs-management-programme/ndmp-natalizumab-protocol.pdf]. Patients who are anti-JCV positive with a titer < 1.5 units, the serological tests are repeated 6-monthly. However, in those with a titer more than 1.5, no further serological testing is recommended [https://www.hse.ie/eng/about/who/acute-hospitals-division/drugs-management-programme/ndmp-natalizumab-protocol.pdf].

Neuroimaging with MRI brain plain is recommended within 3 months before commencing treatment and within 6 months after being on natalizumab. Annual MRI brain are repeated in JCV negative patients, 6-monthly in those with a JCV titer of < 1.5, and 4-monthly in those with a JCV > 1.5 [https://www.hse.ie/eng/about/who/acute-hospitals-division/drugs-management-programme/ndmp-natalizumab-protocol.pdf].

The definite diagnosis requires brain biopsy [19]. The neuroimaging findings coupled with a positive cerebrospinal fluid for JCV-DNA PCR presence is also diagnostic [18]. The characteristic MRI brain features include hyperintense lesions on FLAIR and T2 sequences and hypointense on T1 with or without contrast enhancements and minimal oedema [17].

Immune reconstitution inflammatory syndrome (IRIS) occurs in around 70% of patients who are treated with plasma exchange (PLEX) [18]. Case series of 42 patients with PML due to Nz reported early immunologic rebound may be accelerated due to PLEX which has a poor prognostic value in survival [19]. High-dose steroids are used for counteracting the severe immune response which can pose a serious risk and poor prognosis [20]. For these reasons, we in our patient decided not to use plasma exchange because of his highly active disease activity in the past and likelihood of an accelerated immune response and a poorer outcome.

Cidofovir has been linked to mitigating IRIS rather affecting the JCV directly that has been thought to benefit these patients [21]. Mirtazapine through its effect on serotonergic 5HT2A sites on glial cells which are linked to JCV pathogenesis might also have some benefit [22].

Typically, those with a fatal outcome related to natalizumab-associated PML-IRIS die within 6 months [23]

## Conclusion

- PML is a serious complication of MS treatment which is most commonly related to natalizumab. Clinical features, neuroimaging findings, and cerebrospinal fluid are required to diagnose the condition. Patients who are on natalizumab should be aware and consented for the risk of PML. They should periodically be re-assessed for their relative PML risk; explanation and consenting in those who are on natalizumab with a higher risk should be taken into account for their MS management. There is a growing body of evidence that suggests switching patients from natalizumab who have a higher risk of PML to other safer treatment options.

Future guidelines relating to natalizumab in MS should consider taking into account the following:

- Assessment of the cumulative risk of PML beyond 6 years and risk stratification for those with a high titer of JCV in serum beyond 1.5 and more than 6 years on natalizumab.
- Careful consideration in selecting safer MS therapies in patients who are at a higher risk of PML and potential risks of MS relapse during switching therapies.
- Reinstitution and selection of MS treatment in those with PML and high MS activity.

## Data Availability

All the data available has been shared in this case report.

## Abbreviations

PML: Progressive multifocal leukoencephalopathy
Nz: Natalizumab
DNA: Deoxyribonucleic acid
PCR: Polymerase chain reaction
IRIS: Immune reconstitution inflammatory syndrome
MS: Multiple sclerosis
JCV: John Cunningham virus
